# *LETM1* haploinsufficiency causes mitochondrial defects in cells from humans with Wolf-Hirschhorn syndrome: implications for dissecting the underlying pathomechanisms in this condition

**DOI:** 10.1242/dmm.014464

**Published:** 2014-03-13

**Authors:** Lesley Hart, Anita Rauch, Antony M. Carr, Joris R. Vermeesch, Mark O’Driscoll

**Affiliations:** 1 Human DNA Damage Response Disorders Group, Genome Damage and Stability Centre, School of Life Sciences, University of Sussex, Brighton, BN1 9RQ, UK.; 2 DNA Replication and Cell Cycle Group, Genome Damage and Stability Centre, School of Life Sciences, University of Sussex, Brighton, BN1 9RQ, UK.; 3 University of Zurich, Institute of Medical Genetics, Wagistrasse 12, CH-8952 Schlieren, Switzerland.; 4 Center for Human Genetics, UZ Leuven, Gasthuisberg, Herestraat 49, B-3000 Leuven, Belgium.

**Keywords:** LETM1, Wolf-Hirschhorn syndrome, Mitochondria

## Abstract

Wolf-Hirschhorn syndrome (WHS) represents an archetypical example of a contiguous gene deletion disorder – a condition comprising a complex set of developmental phenotypes with a multigenic origin. Epileptic seizures, intellectual disability, growth restriction, motor delay and hypotonia are major co-morbidities in WHS. Haploinsufficiency of *LETM1*, which encodes a mitochondrial inner-membrane protein functioning in ion transport, has been proposed as an underlying pathomechanism, principally for seizures but also for other core features of WHS, including growth and motor delay. Growing evidence derived from several model organisms suggests that reduced LETM1 expression is associated with some element of mitochondrial dysfunction. Surprisingly, LETM1-dependent mitochondrial functional deficits have not previously been described in cells from individuals with WHS. Here, using a unique panel of WHS-patient-derived cell lines with deletions of differing sizes, incorporating *LETM1* or not, we show, for the first time, that LETM1 expression is reduced in mitochondria isolated from WHS-patient cells. Furthermore, we show that this is associated with distinct mitochondrial phenotypes, including altered intracellular [Ca^2+^] levels, dysfunctional mitochondrial transition-pore opening, hyperpolarization and superoxide leakage from resting mitochondria. Interestingly, we find that these phenotypes segregate with seizures in our WHS cohort. Our findings identify novel cellular phenotypes in WHS attributable to a 50% reduction in LETM1 expression level; these phenotypes could underlie and/or contribute to some of the core clinical features of this condition.

## INTRODUCTION

**Fig. 1:**
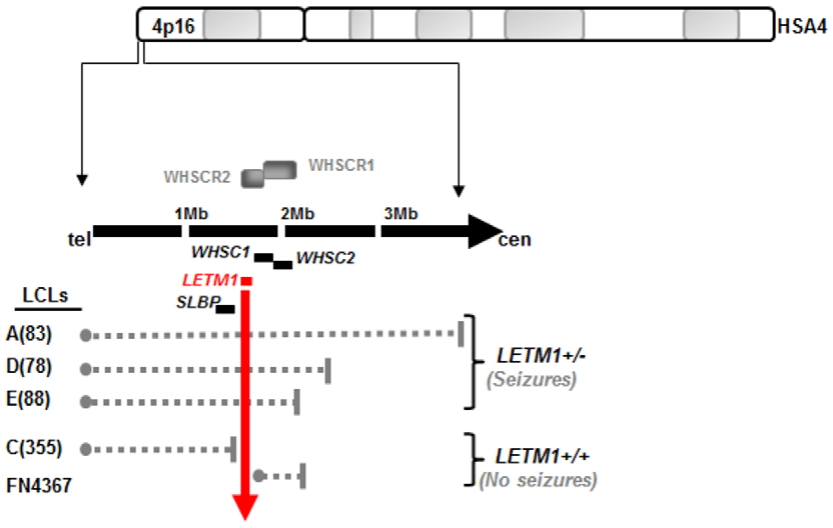
**An ideogram of human somatic autosome (HSA) 4, focusing on 4p16.3 and showing the relative positions of the WHS critical regions WHSCR1 and WHSCR2.** The relative position of certain key genes within and adjacent to these regions are also shown, with *LETM1* highlighted in red. The various LCLs used in this study are listed on the bottom left-hand side adjacent to the dashed grey lines, which depict the position and extent of their specific deletions. The vertical red arrow is to denote which LCLs exhibit *LETM1* haploinsufficiency and which do not. The LCLs A(83), D(78) and E(88) are from patients described in Maas et al. (Maas et al., 2008). Deletion sizes are: A(83), tel-3.85Mb; D(78), tel-2.42Mb; and E(88), tel-2.161Mb. The LCL C(355) has a terminal deletion up to and including *SLBP* and is clinically described in Engbers et al. (Engbers et al., 2009), whereas FN4367 is described in Rauch et al. (Rauch et al., 2001). tel, telomere; cen, centromere.

Wolf-Hirschhorn syndrome (WHS) is a contiguous gene deletion disorder caused by hemizygous deletion within chromosome 4p16.3 (Bergemann et al., 2005; Hirschhorn, 2008). The core clinical features of WHS consist of microcephaly, growth retardation, intellectual disability, atrial and ventricular septal defects, skeletal abnormalities, a characteristic facial dysmorphology, hypotonia, and epileptic seizures (Hirschhorn and Cooper, 1961; Hirschhorn et al., 1965; Wolf et al., 1965). The spectrum and severity of these clinical features typically correlate with deletion size (Battaglia et al., 2008; Maas et al., 2008; Van Buggenhout et al., 2004; Zollino et al., 2000). WHS is generally regarded as a multigenic disorder, although two critical regions have been described: WHSCR1 and WHSCR2, for WHS critical region 1 and 2, respectively (see Fig. 1). These critical regions are based on the demarcation of the minimum region of overlap in individuals exhibiting WHS-like phenotypes. WHSCR1 incorporates part of the WHS candidate gene *WHSC1* and the entire *WHSC2* gene (White et al., 1995; Wright et al., 1997). WHSCR2 incorporates *LETM1* and part of *WHSC1* but not *WHSC2* (Rodríguez et al., 2005; Zollino et al., 2003) (Fig. 1). It is thought that haploinsufficiency of *WHSC1* and *WHSC2* accounts for many of the core phenotypes in WHS.

*WHSC1*, also known as *NSD2* (nuclear receptor SET domain containing) and *MMSET* (multiple myeloma SET domain containing), encodes a putative histone methyltransferase with features of a transcriptional co-repressor (Kim et al., 2008; Marango et al., 2008). WHSC1 (NSD2/MMSET) controls histone H3-trimethylation on lysine-36 (H3-K36-me^3^), a modification associated with actively transcribed regions (Li et al., 2009; Nimura et al., 2009). *WHSC2*, also known as *NELF-A*, encodes a component of the negative elongation factor (NELF) complex (Narita et al., 2007). This complex induces promoter-proximal pausing by inhibiting RNA polymerase II progression early during elongation, thereby altering expression of its target genes in a positive and negative manner (Chiba et al., 2010; Gilchrist et al., 2008). Although haploinsufficiency of both *WHSC1* and *WHSC2* are thought to underlie many of the core clinical features of WHS, other genes additionally contribute to features such as facial dysmorphology, microcephaly and growth retardation, reinforcing the multigenic nature of this disorder (Engbers et al., 2009; Hammond et al., 2012; Hannes et al., 2012; South et al., 2007; South et al., 2008; Van Buggenhout et al., 2004). Untangling the complex functional relationships between the various genes that are haploinsufficient in WHS is essential to fully understand the underlying pathophysiology of this condition.

Over 90% of WHS patients with haploinsufficiency of the typical WHS critical regions exhibit severe generalised tonic-clonic epileptic seizures within the first 3 years of life (Battaglia et al., 2008; Battaglia et al., 2009). This represents a major clinical challenge in these individuals (Battaglia and Carey, 1999). Although generally improving with age, seizures can also be managed with valproate and/or phenobarbital (Battaglia et al., 2009). Haploinsufficiency of *LETM1*, which encodes the mitochondrial protein leucine-zipper EF-hand-containing transmembrane protein 1 (LETM1), is thought to contribute to seizure development in WHS (Dimmer et al., 2008; Endele et al., 1999; Hasegawa and van der Bliek, 2007; McQuibban et al., 2010; Nowikovsky et al., 2004; Tamai et al., 2008). This contribution is largely based on precisely defining the location and content of overlapping deletions in WHS and WHS-like patients with and without seizures. Nevertheless, there is additional evidence to suggest that other genes, aside from *LETM1*, might also play a role in some cases (Andersen et al., 2013; Misceo et al., 2012; South et al., 2007).

**TRANSLATIONAL IMPACT**

**Clinical issue**

Wolf-Hirschhorn syndrome (WHS) is a genetic disorder that is caused by contiguous hemizygous deletions (deletions that affect only one copy of a chromosome pair) within chromosome 4p16.3. It is a complex disorder but its core clinical features consist of microcephaly, growth restriction, intellectual disability, cardiac and skeletal abnormalities, facial dysmorphology, hypotonia and epileptic seizures. The presence of only one functional copy of specific genes located in chromosome 4p16.3 (haploinsufficiency) has been implicated in the pathogenesis of this condition. To fully understand WHS, it is necessary to untangle the complex relationships between the size of the hemizygous deletion and the spectrum and severity of the condition’s clinical features. Haploinsufficiency of *LETM1*, which encodes a mitochondrial protein that is involved in ion transport, is thought to underlie the seizures and some other features of classical WHS, but how *LETM1* haploinsufficiency contributes to seizure presentation or indeed whether individuals with WHS exhibit any LETM1-dependent phenotypes has not been fully elucidated.

**Results**

In this study, the authors use a unique panel of cell lines derived from individuals with WHS to show that LETM1 expression is reduced in cell extracts and mitochondria isolated from those with *LETM1* haploinsufficiency. They catalogue several interdependent newly identified mitochondrial phenotypes that are associated with reduced LETM1 expression, including altered intracellular calcium levels, dysfunctional mitochondrial transition pore opening, hyperpolarisation and superoxide leakage. Using siRNA and cDNA transfection approaches, they show that these phenotypes are a direct consequence of reduced LETM1 expression. Finally, the authors report that the LETM1-dependent mitochondrial phenotypes segregate with seizures in the small cohort of patients from whom the cell lines were derived.

**Implications and future directions**

These findings provide the first direct evidence that cells with *LETM1* haploinsufficiency obtained from individuals with WHS exhibit reduced LETM1 expression and that mitochondrial dysfunction is a consequence of the 50% reduction in LETM1 expression, thereby providing new pathomechanistic insights into WHS. Although studies in mouse models that have reduced *Letm1* expression strongly suggest a link between reduced Letm1 expression and seizures, recent work in individuals with WHS suggests that *LETM1* haploinsufficiency might also contribute to growth restriction, feeding difficulties, and motor and speech delays; the mitochondrial dysfunctions revealed here could conceivably contribute to these other characteristic features of WHS. Finally, these novel findings raise the possibility that mitochondrial-based therapeutic interventions could be of benefit in the management of WHS and should therefore be investigated.

LETM1 is a highly attractive contributing candidate for seizures in WHS principally because of its demonstrated role in various aspects of mitochondrial homeostasis (Dimmer et al., 2008; Endele et al., 1999; Hasegawa and van der Bliek, 2007; McQuibban et al., 2010; Nowikovsky et al., 2004; Tamai et al., 2008). Impaired mitochondrial function, abnormal ion-buffering and elevated mitochondrially derived reactive oxygen species (ROS) are all established interrelated pathomechanisms of seizures (Folbergrová and Kunz, 2012; Kang et al., 2013). Reduced expression of *LETM1* and its orthologues have variously been identified as impacting upon mitochondrial morphology (increased swelling and elongation) and consequently upon function by controlling K^+^/H^+^ ion exchange, and also upon mitochondrial Ca^2+^ buffering by acting as a Ca^2+^/H^+^ antiporter (Jiang et al., 2009; Nowikovsky et al., 2012).

The precise role of LETM1 in mitochondrial ion transport is currently an area of intense debate (Nowikovsky et al., 2012; Nowikovsky et al., 2009). Nevertheless, recent studies modelling reduced *Letm1* expression in mice and rats demonstrated a reduced threshold for chemically induced seizures (Jiang et al., 2013; Zhang et al., 2013). Furthermore, in the *Letm1* rat study, the authors reported increased mitochondrial swelling and decreased mitochondrial cytochrome *b* levels (Zhang et al., 2013). In the mouse study, an associated impairment in mitochondrial Ca^2+^ buffering and consequent mitochondrial function were documented in *Letm1*^+/−^ animals (Jiang et al., 2013).

Surprisingly, specific LETM1-dependent functional mitochondrial defects have not been demonstrated previously in WHS-patient-derived cell lines. In fact, in one study, although reduced LETM1 expression was observed in WHS-patient-derived fibroblasts, levels were found to be unaffected in the corresponding patient lymphoblastoid cell lines (LCLs) (Dimmer et al., 2008). Here, using a unique panel of WHS-patient-derived LCLs from individuals with deletions either incorporating *LETM1* or not, we set out to identify and characterise LETM1-dependent mitochondrial phenotypes.

### RESULTS

The patient LCLs used in this study and their respective deletions are shown in Fig. 1. All of these lines are derived from patients previously described in the literature (Engbers et al., 2009; Hannes et al., 2010; Maas et al., 2008; Rauch et al., 2001). Of note, FN4367 is an LCL derived from the patient described by Rauch and colleagues involving one of the smallest deletions catalogued in 4p16.3 in a patient exhibiting mild WHS phenotypes, but without seizures (Rauch et al., 2001). This is an interstitial 191.5 kb deletion encompassing WHSCR1, and not involving *LETM1* (Rauch et al., 2001). LCL C(355) is from a WHS-like individual with a terminal deletion up to and including *SLBP*, thereby preserving *LETM1* and the WHSCRs (Engbers et al., 2009; Hannes et al., 2010). This individual has not presented with seizures (Engbers et al., 2009).

### WHS-patient LCLs haploinsufficient for *LETM1* exhibit reduced LETM1 expression

**Fig. 2:**
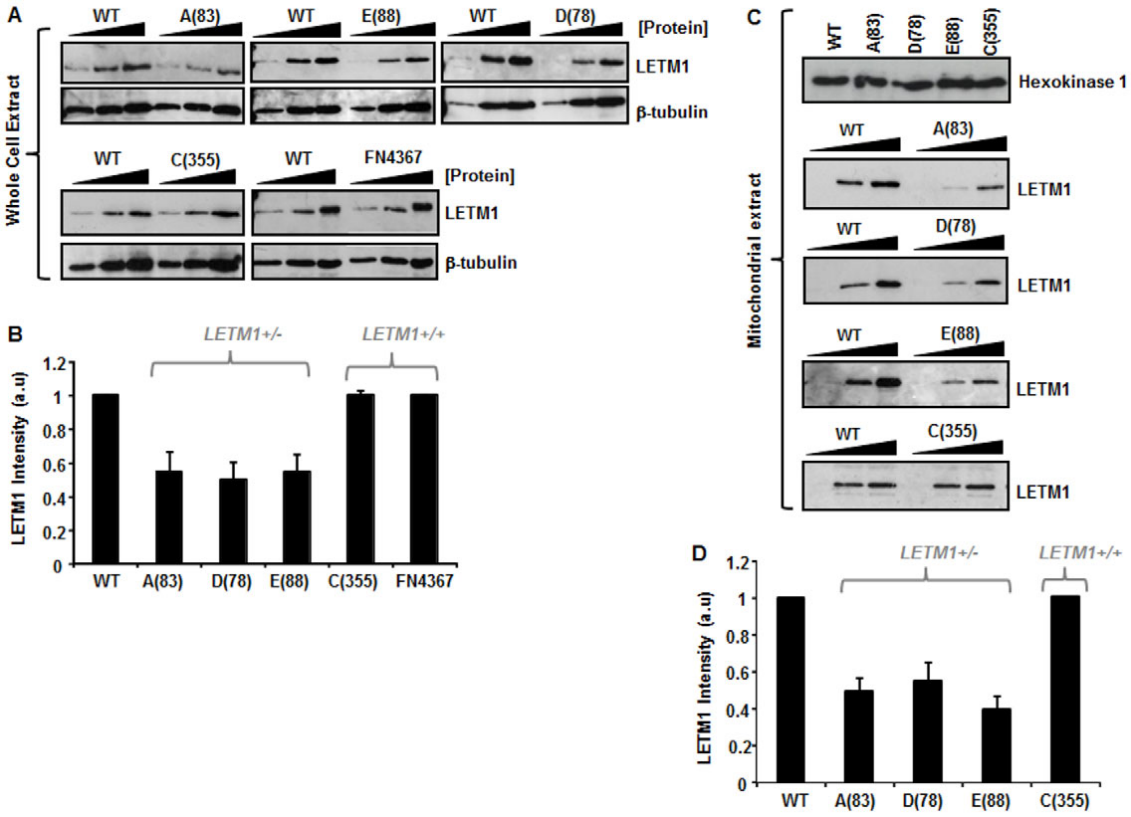
**LETM1 expression in WHS-patient LCLs.** (A) Urea-based whole cell extracts (WCEs) were titrated (2.5, 5, 10 μg) for each WHS-patient LCL and run alongside those from wild-type (WT) control extracts from a normal individual and blotted for LETM1 expression. Membranes were re-probed for β-tubulin to confirm loading. (B) LETM1 expression for each WHS cell line relative to WT were quantified (a.u., arbitrary units) using ImageJ software from scanned blots. Each quantitation represents the mean ± s.d. from at least three separate determinations. Measurements were also taken directly from membranes during enhanced chemiluminescence (ECL) development using the Image Quant LAS 4000 luminescent image analyser and analysed with Image Quant TL7.01 software, which yielded identical comparative changes as ImageJ processing. A(83), D(78) and E(88) exhibit reduced LEMT1 expression compared with WT, C(355) and FN4367 (*P*<0.05, Student’s *t*-test). (C) Urea-extracts were prepared from anti-TOM22 affinity-isolated mitochondria using the Miltenyi Biotech magnetic capture kit. Hexokinase expression, as a mitochondrial marker, is shown for each LCL. Different amounts of urea-mitochondrial extract was subjected to western blot analysis for LETM1 expression. (D) Relative LETM1 expression of each line compared with WT were quantified using ImageJ software from scanned blots as in B. Each quantitation represents the mean ± s.d. from at least three separate determinations. A(83), D(78) and E(88) exhibit reduced LEMT1 expression compared with WT and C(355) (*P*<0.05, Student’s *t*-test).

Surprisingly, reduced LETM1 expression has not previously been consistently and unequivocally demonstrated in WHS-patient-derived cells despite *LETM1* haploinsufficiency being proposed to contribute to seizure development in this condition (Dimmer et al., 2008; Hasegawa and van der Bliek, 2007). To conclusively address this issue, we interrogated LETM1 expression in our cohort of genomically characterised WHS and WHS-like patient LCLs. Using urea-denatured whole-cell extracts (WCEs) prepared from our panel of patient LCLs with and without deletions incorporating *LETM1*, we found that haploinsufficiency of *LETM1* is in fact associated with an ~50% reduction in LETM1 expression (Fig. 2A,B). LCLs derived from patients A(83), D(78) and E(88) all showed reduced LETM1 expression associated with *LETM1* haploinsufficiency. This is in contrast to extracts prepared from C(355) and FN4367, two lines with normal *LETM1* copy number (Fig. 2A,B).

LETM1 protein resides in the inner mitochondrial membrane (IMM) (Endele et al., 1999; Tamai et al., 2008). Considering our results obtained using urea-denatured WCEs, we reasoned that WHS-patient LCLs haploinsufficient for *LETM1* would also exhibit reduced LETM1 expression in mitochondria. Using an anti-TOM22 affinity-based magnetic mitochondrial isolation system (Miltenyi Biotech), we isolated intact mitochondria from each of our WHS-patient LCLs. Similar to our results obtained from WCEs, we found that mitochondrial extracts prepared from LCLs from patients with *LETM1* haploinsufficiency exhibited reduced LETM1 compared with patient-derived LCLs with normal *LETM1* copy number (Fig. 2C,D). Therefore, we conclude that *LETM1* haploinsufficiency in the context of WHS is consistently associated with reduced expression of LETM1 in mitochondria of patient LCLs.

In our patient cohort, reduced mitochondrial LETM1 expression seems to segregate with seizure development. To try and understand whether we could link LETM1 expression levels with a specific mitochondrial deficit or a set of deficits, we investigated different aspects of mitochondrial function in these patient LCLs.

### *LETM1* haploinsufficiency in WHS does not affect mitochondrial mass but is associated with elevated intracellular [Ca^2+^]

**Fig. 3:**
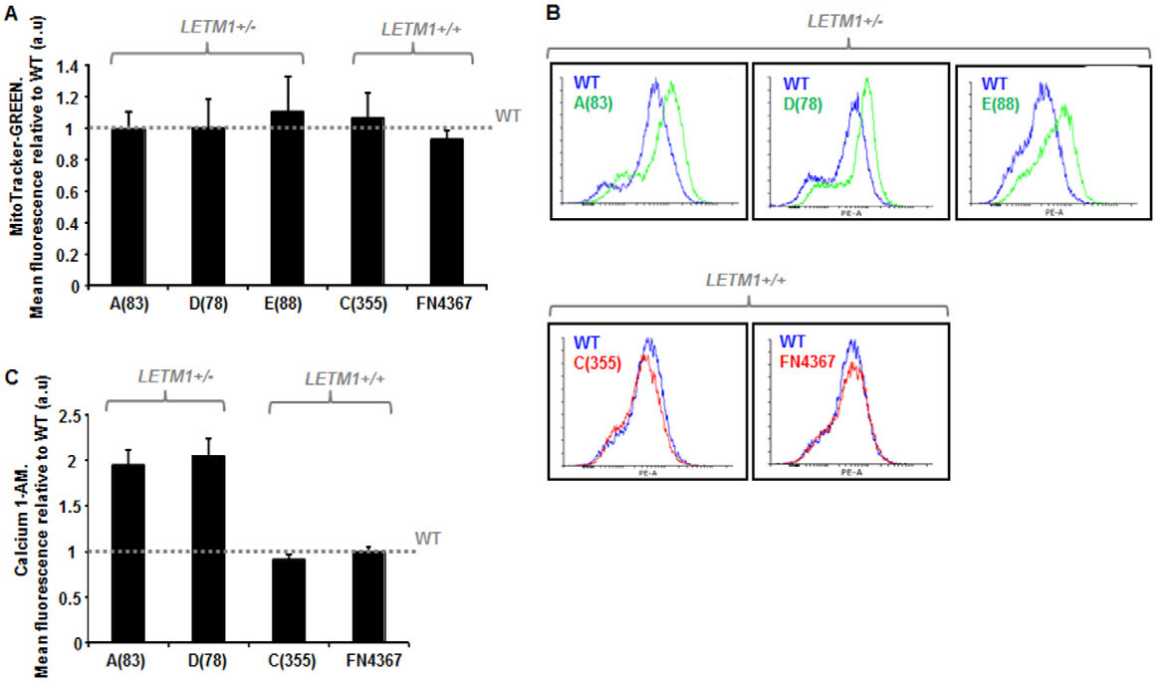
**Mitochondrial mass and intracellular [Ca^2+^] in WHS-patient LCLs.** (A) Mean relative fluorescence of MitoTracker-Green in WHS LCLs is shown relative to that of wild-type (WT) normal LCLs, the latter illustrated by the horizontal dashed line. Cells were treated with 250 nM MitoTracker-Green for 15 minutes and fluorescence quantified using the FACS Canto platform (FITC-A: area under the curve). All of the WHS LCLs, irrespective of *LETM1* copy number, exhibit a comparable level of MitoTracker-Green fluorescence compared with WT. Data represents the mean ± s.d. from three separate experiments. (B) Calcium 1-AM profiles for the WHS LCLs. The profile for the wild-type (WT) normal LCLs is in blue. WHS LCLs with *LETM1* haploinsufficiency are in green, whereas those with normal LETM1 copy number are depicted in red. (C) Mean relative Calcium 1-AM levels in WHS LCLs is shown relative to that of wild-type (WT) normal LCLs, the latter illustrated by the horizontal dashed line. Cells were treated with 10 μM Calcium 1-AM for 20 minutes and fluorescence analysed (excitation/emission 506/531) on the FACS Canto platform. Both A(83) and D(78) LCLs exhibit an elevated level of Calcium 1-AM relative to WT LCLs and the WHS LCLs C(355) and FN4367 (*P*<0.05, Student’s *t*-test). Both A(83) and D(78) exhibit *LETM1* haploinsufficiency, whereas C(355) and FN4367 have normal *LETM1* copy number. Data represents the mean ± s.d. from three separate experiments (FITC-A: area under the curve).

Using MitoTracker-Green to determine mitochondrial mass, we found that all of the WHS-patient LCLs exhibited a similar mitochondrial content to each other, irrespective of *LETM1* copy number, and to LCLs from a normal wild-type (WT) control (Fig. 3A). Therefore, haploinsufficiency of *LETM1*, although resulting in reduced mitochondrial LETM1 expression, does not seem to affect mitochondrial content within WHS-patient LCLs.

Mitochondria represent one of the key cellular Ca^2+^ buffering systems within the cell (Osellame et al., 2012; Szabadkai and Duchen, 2008). Because LETM1 has been described as a Ca^2+^/H^+^ antiporter, we investigated levels of intracellular Ca^2+^ using the fluorescent long-wave Ca^2+^ indicator, Calcium 1-AM, in the context of *LETM1* haploinsufficiency (Jiang et al., 2009). Interestingly, we found elevated levels of intracellular [Ca^2+^] in WHS-patient LCLs with *LETM1* haploinsufficiency [i.e. A(83), D(78) and E(88)], compared with those with normal *LETM1* copy number [i.e. C(355) and FN4367], potentially suggestive of a problem in Ca^2+^ buffering in these cells (Fig. 3B,C). This phenotype has not previously been described in WHS-patient cells, but is consistent with reduced mitochondrial Ca^2+^ uptake recently demonstrated in cells from a *Letm1^+/−^* mouse model (Jiang et al., 2013).

### *LETM1* haploinsufficiency in WHS is associated with impaired mPTP dynamics

**Fig. 4:**
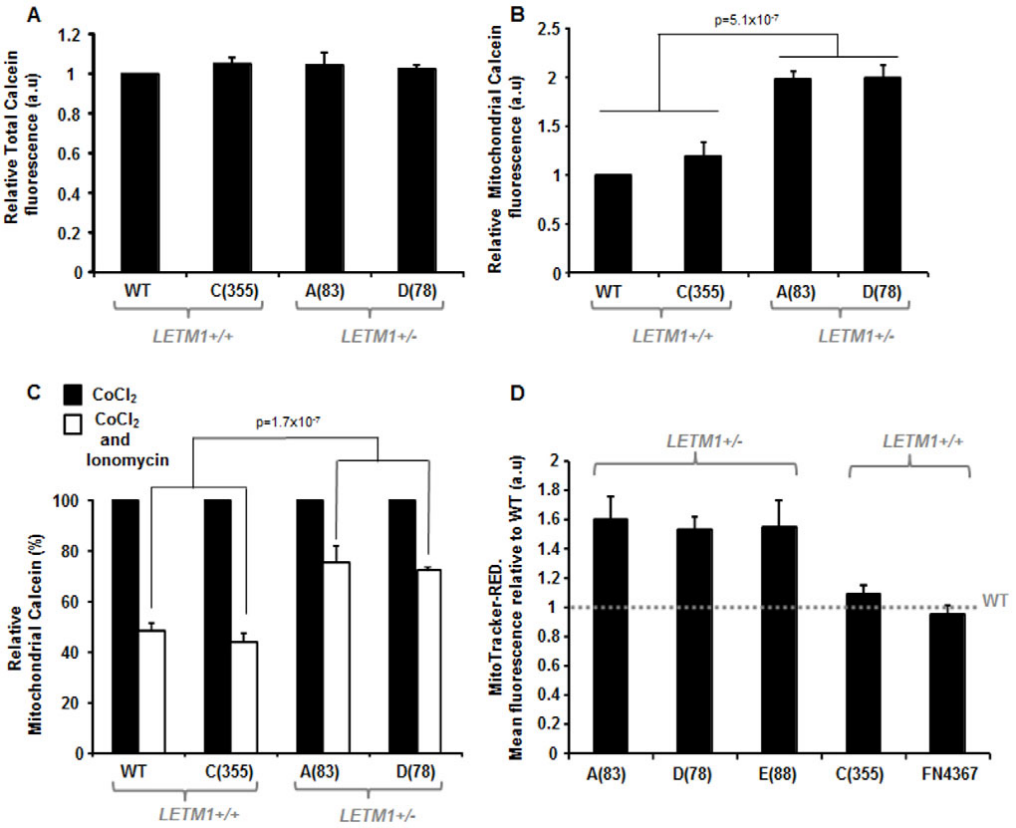
**Mitochondrial transition pore (mPTP) dyamics and mitochondrial membrane polarization in WHS-patient LCLs.** (A) Total cellular Calcein fluorescence is comparable between WT LCLs and WHS patient and WHS-like patient LCLs irrespective of LETM1 copy number. Calcein fluorescence was measured in the absence of CoCl_2_ and expressed as fold change relative to WT LCLs (a.u., arbitrary units). Data represents the mean ± s.d. from three separate experiments (FITC-A: area under the curve). (B) Mitochondrial calcein fluorescence levels (a.u., arbitrary units) indicate that the WHS LCLs A (83) and D(78) both exhibit elevated levels of mitochondrial calcein retention compared with WT LCLs and those of patient C(355). Here, calcein fluorescence is measured in the presence of CoCl_2_, which quenches the cytoplasmic (i.e. non-mitochondrially derived) signal. Data represent the mean ± s.d. from three separate experiments (FITC-A: area under the curve). (C) Mean relative level of mitochondrial calcein retention in WHS LCLs of differing *LETM1* copy number compared with WT LCLs in the absence (black bar) or presence (white bar) of the Ca^2+^ ionophore, ionomycin. Both WT and C (355) exhibit a near 60% drop in calcein retention following ionomycin (500 nM) relative to CoCl_2_ treatment alone. This is in contrast to A(83) and D(78) LCLs, which only drop by about 20–30% under these conditions. This is suggestive of an insensitive mPTP favouring a closed conformation conducive to mitochondrial calcein retention. Data represents the mean ± s.d. from at least three separate experiments (FITC-A: area under the curve). (D) Mean relative fluorescence of MitoTracker-Red in WHS LCLs is shown relative to that of wild-type (WT) normal LCLs, the latter illustrated by the horizontal dashed line. Cells were treated with 250 nM MitoTracker-Red for 15 minutes and fluorescence quantified using the FACS Canto platform. WHS LCLs A(83), D(78) and E(88) exhibit elevated MitoTracker-Red fluorescence, suggestive of hyperpolarised resting mitochondria (elevated ΔΨ_mito_), compared with WT LCLs and those from patients C(355) and FN4367 (*P*<0.05, Student’s *t*-test). Data represent the mean ± s.d. from three separate experiments (PE-A: area under the curve). Control experiments, using depolarising and hyperpolarising treatments, are shown in supplementary material Fig. S1A,B.

The mitochondrial permeability transition pore (mPTP) is a non-selective voltage-dependent mitochondrial channel proposed to reside in the IMM (Bernardi, 2013; Siemen and Ziemer, 2013). Opening of the mPTP increases the permeability of the IMM to solutes of >1.5 kDa in size. Under normal physiological conditions, this pore flickers between an open and closed state. However, various stimuli, such as elevated ROS levels and increased levels of mitochondrial calcium, promote the sustained opening of this pore. We investigated mPTP dynamics using the MitoProbe Transition Pore Assay Kit (Invitrogen-MitoProbes), which uses calcein fluorescence in the presence of CoCl_2_ as an indicator of the retention of calcein within mitochondria when the mPTP is in the closed state; CoCl_2_ quenches cytoplasmic calcein fluorescence, rendering the signal mitochondrial-specific. Firstly, we found that total calcein fluorescence load (i.e. in the absence of CoCl_2_) was comparable between all the patient-derived LCLs and WT cells (Fig. 4A). Interestingly, we found that WHS LCLs from patient A(83) and D(78) specifically exhibited elevated mitochondrial calcein fluorescence compared with WT LCLs and those from patient C(355) (Fig. 4B). This is suggestive of the mPTP favouring a predominantly closed state and/or insensitive ability to transition between open and closed conformations in resting mitochondria in A(83) and D(78) LCLs. These cell lines both exhibited *LETM1* haploinsufficiency and decreased mitochondrial LETM1 levels compared with WT LCLs and those of patient C(355) (Figs 1, 2).

Treatment of cells with a Ca^2+^ ionophore such as ionomycin induces mitochondrial Ca^2+^ overload, typically resulting in mPTP opening and consequent reduction of calcein retention. Treatment of WT LCLs and LCLs from patient C(355) with ionomycin resulted in an ~60% decrease in mitochondrial calcein fluorescence, consistent with mPTP opening, as expected (Fig. 4C). In stark contrast, A (83) and D(78) patient-derived LCLs exhibited only a modest 25–30% relative reduction in mitochondrial calcein fluorescence under identical treatment conditions (Fig. 4C). These results are consistent with a [Ca^2+^]-insensitive mPTP adopting a predominantly closed conformation in WHS-patient LCLs with *LETM1* haploinsufficiency, further indicative of altered mitochondrial-mediated [Ca^2+^] buffering in this context. This cellular phenotype has not previously been described in WHS-patient cells.

### *LETM1* haploinsufficiency in WHS is associated with mitochondrial membrane hyperpolarisation: *elevated* ΔΨ_mito_

The mitochondrial membrane potential (ΔΨ_mito_) is exquisitely sensitive to mitochondrial ion flux. In their identification of LETM1 as a mitochondrial Ca^2+^/H^+^ antiporter, Jiang and colleagues found that siRNA-mediated silencing of *Letm1* in *Drosophila* S2 cells resulted in a modest although notable (~1.3-fold) increase in ΔΨ_mito_ (Jiang et al., 2009). Whether only a 50% reduction in LETM1 expression as a consequence of *LETM1* haploinsufficiency could result in a similar effect upon ΔΨ_mito_ has not previously been investigated. Considering our findings regarding intracellular [Ca^2+^] and mPTP (Figs 3, 4), we examined ΔΨ_mito_ status in resting mitochondria in LCLs from our WHS cohort. Using MitoTracker Red-CMXRos fluorescence, we consistently found spontaneously elevated ΔΨ_mito_ (~1.4- to 1.6-fold) in WHS-patient LCLs with *LETM1* haploinsufficiency [i.e. A(83), D(78), E(88)] compared with those with normal LETM1 expression [i.e. C(355), FN4367] and with WT LCLs (Fig. 4D). This suggests that *LETM1* haploinsufficiency in the context of WHS is associated with spontaneous hyperpolarisation of resting mitochondria. This represents an additional novel mitochondrial phenotype associated with WHS and, in the context of this cohort, an additional phenotype segregating with seizure expression.

### *LETM1* haploinsufficiency in WHS is associated with elevated mitochondrial superoxide (O_2_^−^) production

**Fig. 5:**
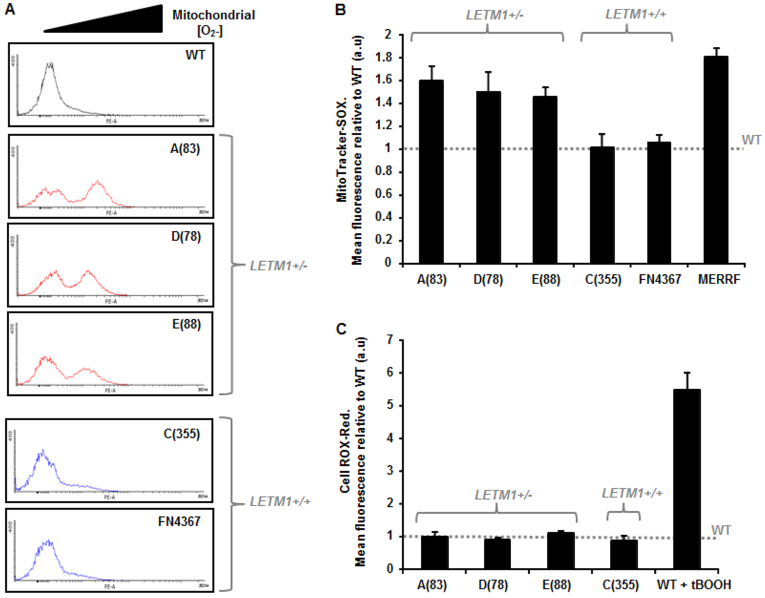
**Mitochondrial [O_2_^−^] and total cellular ROS levels in WHS-patient LCLs.** (A) Representative fluorescence profiles following treatment of WT and WHS-patient LCLs with MitoTracker-SOX (250 nM for 15 minutes) as obtained from the FACS Canto platform. Elevated levels of mitochondrial-derived ROS (O_2_^−^) are indicated by the black triangle. The WHS LCLs with *LETM1* haploinsufficiency show marked peaks towards the right of each profile (elevated fluorescence) compared with WT LCLs and those from the WHS patients with normal *LETM1* copy number [C(355) and FN4367]. A control experiment using thalidomide, a drug known to produce reactive oxygen species, is shown in supplementary material Fig. S1C. (B) Mean relative fluorescence of MitoTracker-SOX in WHS LCLs is shown relative to that of wild-type (WT) normal LCLs, the latter illustrated by the horizontal dashed line. WHS LCLs A(83), D(78) and E(88) exhibit elevated MitoTracker-SOX fluorescence indicative of elevated O_2_^−^ production from resting mitochondria, compared with WT LCLs (dashed line) and those from patients C(355) and FN4367 (*P*<0.05, Student’s *t*-test). Furthermore, a MERRF (myoclonus epilepsy associate with ragged-red fibres) patient-derived LCL also exhibited a similarly significantly elevated MitoTracker-SOX fluorescence to that of the *LETM1^+/−^* WHS LCLs. Data represent the mean ± s.d. from three separate experiments (PE-A: area under the curve). (C) Mean relative fluorescence of CellROX-Red in WHS LCLs is shown relative to that of wild-type (WT) normal LCLs, the latter illustrated by the horizontal dashed line. CellROX-Red fluorescence is a measure of total cellular ROS. Pre-treatment of WT LCLs with the oxidant *tert*-Butyl hydroperoxide (tBOOH; 100 μM, 1 hour) resulted in an ~fivefold increase in CellROX-Red mean fluorescence relative to untreated WT LCLs. All of the WHS LCLs, irrespective of *LETM1* copy number, exhibit a comparable level of CellROX-Red fluorescence compared with WT. Data represent the mean ± s.d. from three separate experiments (PE-A: area under the curve).

Mitochondrial dysfunction is often associated with elevated superoxide (O_2_^−^) leakage (Kang and Pervaiz, 2012; Pieczenik and Neustadt, 2007). Using MitoTracker-SOX fluorescence as a direct measure of mitochondrial [O_2_^−^], we found a striking increase (~1.4-to 1.6-fold) in mitochondrial ROS in WHS-patient LCLs with *LETM1* haploinsufficiency, specifically A(83), D(78) and E(88) patient LCLs, in contrast to WHS-patient cells with normal *LETM1* copy number [i.e. C(355), FN4367] and to WT LCLs (Fig. 5A,B). In fact, the elevated level of mitochondrial O_2_^−^ production in these LCLs was comparable to that of an LCL derived from a patient with myoclonus epilepsy with ragged-red fibres (MERRF) (Fig. 5B). MERFF is a primary mitochondrial disorder. Interestingly, elevated mitochondrially derived O_2_^−^ was not associated with an overall elevated level of intracellular ROS in these cells, as determined by CellROX-Red fluorescence (Fig. 5C). These data further indicate that *LETM1* haploinsufficiency in the context of WHS is associated with specific mitochondrial dysfunctions.

Our analysis of WHS-patient LCLs has shown that haploinsufficiency of *LETM1* is associated with reduced LETM1 expression within isolated mitochondria and this segregates with elevated intracellular [Ca^2+^], an insensitive mPTP adopting a predominantly closed conformation, spontaneously elevated ΔΨ_mito_ indicative of hyperpolarized mitochondria, as well as elevated mitochondrial ROS production in resting mitochondria. These represent novel cellular phenotypes for WHS. Interestingly, these phenotypes also seem to segregate with the WHS patients within our small cohort who have presented with seizures. Mindful of the fact that other genes are also deleted in our WHS lines and that other regions within 4p16.3 might also contribute to seizure development in this condition, we set out to establish whether reduced expression of LETM1 alone could recapitulate some of the key mitochondrial phenotypes that we have identified here.

### Reduced LETM1 is associated with elevated ΔΨ_mito_ and elevated mitochondrial O_2_^−^

**Fig. 6:**
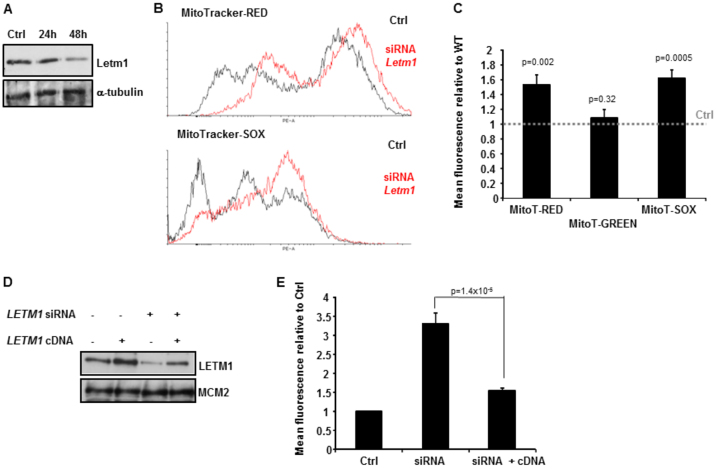
**Knockdown of *LETM1* recapitulates mitochondrial phenotypes of WHS-patient LCLs with haploinsufficiency of *LETM1***. (A) Western blot analysis of Letm1 expression following siRNA-mediated silencing of *Letm1* in mouse Neuro2A cells. An ~50% reduction in Letm1 was observed 48 hours post-transfection. Ctrl, control-transfected. The membrane was re-probed for α-tubulin to confirm loading. (B) Representative fluorescence profiles following siRNA-mediated reduction of *Letm1* to 50% in Neuro2A cells and incubation with MitoTracker-Red (upper panel) and MitoTracker-SOX (lower panel). The siRNA-treated profile for each MitoTracker is shown in red. Ctrl, control-transfected. Elevated fluorescence for each MitoTracker is indicated by a rightward shift in the profiles following siRNA of *Letm1* relative to Ctrl. (C) Mean relative fluorescence of MitoTracker-Red, MitoTracker-Green and MitoTracker-SOX levels following siRNA in Neuro2A cells is shown relative to that of control-transfected (Ctrl) cells, the latter illustrated by the horizontal dashed line. The siRNA-mediated reduction of Letm1 to 50% results in elevated MitoTracker-Red and MitoTracker-SOX fluorescence specifically, not impacting upon MitoTracker-Green levels. Therefore, siRNA of *Letm1* is associated with increased hyperpolarisation (elevated ΔΨ_mito_) and elevated O_2_^−^ production from resting mitochondria under these conditions, without impacting upon mitochondrial mass. Data represent the mean ± s.d. from three separate experiments (MitoT-Red and MitoT-Green; PE-A: area under the curve; MitoT-Green: FITC-A: area under the curve). (D) Western blot analysis for LETM1 from human T98G glioblastoma cells following either siRNA of *LETM1* using a 3′UTR-directed oligonucleotide or transfection with a plasmid containing *LETM1*, or a combination of both as indicated. The membrane was re-probed for MCM2 to confirm loading. (E) Mean relative fluorescence of MitoTracker-SOX in T98G cells is shown relative to that of control-transfected cells (Ctrl), following silencing of *LETM1* (siRNA), along with co-transfection with an siRNA-resistant *LETM1* cDNA (siRNA + cDNA). Silencing of LETM1 using a 3′UTR-directed oligonucleotide directed against the endogenous gene results in a marked increase in MitoTracker-SOX levels indicative of elevated mitochondrial ROS production (Ctrl versus siRNA *P*<0.05, Student’s *t*-test). Co-transfection with an siRNA-resistant *LETM1*-encoding plasmid reduces the level of MitoTracker-SOX fluorescence to that of control-transfected cells (Ctrl). Data represent the mean ± s.d. from three separate experiments (PE-A: area under the curve).

Using the mouse neuroblastoma cell line Neuro2A as a model system, we investigated whether siRNA-mediated reduction of *Letm1* alone could recapitulate some of the novel mitochondrial phenotypes we have described here in *LETM1*-haploinsufficient WHS-patient LCLs. We carefully optimised our siRNA procedure (by titration) so as to only obtain an ~50% reduction in Letm1 to closer mimic the WHS-patient LCL situation. As shown in Fig. 6A, siRNA-mediated reduction of Letm1 to ~50% of control-transfected (Ctrl) Neuro2A cells resulted in elevated ΔΨ_mito_ (MitoTracker-Red) and mitochondrial O_2_^−^ production (MitoTracker-SOX), whilst not affecting mitochondrial mass (MitoTracker-Green) (Fig. 6B,C). These phenotypes are identical to those identified here for WHS-patient LCLs haploinsufficient for *LETM1* (Fig. 3A; Fig. 4C; Fig. 5A,B).

To further reinforce the fact that the elevated levels of mitochondrial O_2_^−^ observed under these conditions were directly attributable to reduced LETM1 levels, we performed a similar analysis, although in this case using the human T98G glioblastoma line, with the additional element of complementation with an siRNA-resistant cDNA encoding *LETM1*. The siRNA oligonucleotide was designed to the 3′UTR of *LETM1* and, as expected, resulted in a significant reduction in LETM1, which was also associated with elevated mitochondrial O_2_^−^ (MitoTracker-SOX) (Fig. 6D,E). In fact, under these conditions we observed an over threefold increase in mitochondrial O_2_^−^ production (Fig. 6E).Importantly, co-transfection of the 3′UTR-directed oligo with the siRNA-resistant cDNA was associated with the consequent reduction in mitochondrial ROS (Fig. 6E). These data show that modestly reduced expression of LETM1 alone can cause mitochondrial dysfunction. Unfortunately, we were unable to generate a viable and stable WHS-patient LCL stably transduced with *LETM1* from a lentivirus (CMV-driven pReceiver-Lv105 expression clone LP-W0230-Lv105-0200-S, Genecopoea).

In summary, using a panel of WHS and WHS-like patient LCLs with differing sized deletions incorporating *LETM1* or not, we show that haploinsufficiency of *LETM1* is associated with reduced LETM1 expression and a series of mitochondrial phenotypes. These include elevated intracellular [Ca^2+^], an insensitive mPTP and hyperpolarized mitochondria (elevated ΔΨ_mito_) with elevated mitochondrial O_2_^−^ production. Many of these mitochondrial deficits have been previously associated with seizures. In our patient cohort, these mitochondrial phenotypes segregate with seizure presentation. Our data strongly suggest that a 50% reduction in LETM1 expression as a consequence of *LETM1* haploinsufficiency in WHS can result in overt mitochondrial phenotypes.

## DISCUSSION

WHS is generally regarded as a multigenic condition with haploinsufficiency of the critical regions thought to explain many of the core clinical features, although other genes clearly have additional contributions (Engbers et al., 2009; South et al., 2007; South et al., 2008; Van Buggenhout et al., 2004). For example, we recently identified delayed S-phase progression and impaired chromatin remodelling in WHS-patient LCLs attributable to haploinsufficiency of *SLBP* and/or *WHSC2* (*NELF-A*); phenotypes with implications for the maintenance of epigenetic memory, expansion of stem cell niches, and possibly microcephaly and growth retardation (Kerzendorfer et al., 2012). Assessing the contribution of haploinsufficiency of a single gene towards a specific phenotype or set of phenotypes characteristic of a contiguous gene deletion disorder can be complex. This needs to be considered in the context of variable expressivity, incomplete penetrance or the revelation of recessive alleles and/or complex positional effects (reviewed in Hart and O’Driscoll, 2013). Single-gene contributions are usually based on the existence of patients with rare atypical and/or very small deletions being placed in context of the more commonly sized deletions. Additionally, in some cases murine models might be available, enabling interpretation of the pathomechanistic impact of impaired function of a particular gene. For WHS, several mouse models for genes within human somatic autosome (HSA) 4p16.3 have been used as supportive evidence for the haploinsufficiency of certain genes in underlying specific clinical features of the condition. Examples include *Whsc1* for cardiac and midline abnormalities, *Fgfr3* and *Ctbp1* for skeletal abnormalities, and *Fgfr3*, *Ctbp1*, *Tacc3* and *Hspx153* for growth retardation (Abrams and Jiao, 2009; Näf et al., 2001; Nimura et al., 2009; Simon and Bergemann, 2008).

When considering seizures in WHS, the situation is highly complex. The segregation of seizures with *LETM1* haploinsufficiency does not seem to be absolute (Andersen et al., 2013; South et al., 2008). There are a modest although growing number of cases with sub-telomeric deletions not involving *LETM1* that have been reported with seizures, whereas, conversely, there are also deletion cases incorporating *LETM1* that are reported to be seizure free (Andersen et al., 2013; Bayindir et al., 2013; Engbers et al., 2009; Faravelli et al., 2007; Izumi et al., 2010; Misceo et al., 2012; South et al., 2008; Van Buggenhout et al., 2004). In fact, whether *LETM1* haploinsufficiency plays any role whatsoever in seizure development has been questioned by some (Bayindir et al., 2013; Luo et al., 2011). Importantly, to our knowledge, neither LETM1 expression nor functional mitochondrial characterisation has been investigated in these situations (Bayindir et al., 2013; Luo et al., 2011). Furthermore, *CTBP1* and/or *CPLX1* haploinsufficiency, two genes telomeric to *LETM1*, have also been proposed to possibly underlie seizures in WHS, although, again, without any supportive, associative functional cellular characterization in WHS-patient cell lines (Misceo et al., 2012; Simon and Bergemann, 2008).

Attributing reduced *LETM1* expression as the cause of or contributing factor to seizures in WHS has evolved from a combination of (i) cataloguing patients with different sized deletions and variable seizure expression, (ii) realising LETM1’s mitochondrial localisation and its role in ion transport, and (iii) the overt mitochondrial morphological changes observed in *Saccharomyces cerevisiae*, *Drosophila*, *Caenorhabditis elegans* and even HeLa cells, when LETM1 levels were reduced (Dimmer et al., 2008; Endele et al., 1999; Hasegawa and van der Bliek, 2007; McQuibban et al., 2010; Nowikovsky et al., 2004; Tamai et al., 2008). Furthermore, (iv) downregulation of CG4589, the *LETM1* orthologue in *Drosophila*, also leads to reduced synaptic neurotransmitter release (McQuibban et al., 2010).

Recently, two interesting murine-based models of reduced Letm1 expression have further strengthened the pathomechanistic link between LETM1-induced mitochondrial dysfunction and seizures. Zhang and colleagues showed that stereotaxic intra-hippocampal injection of a *Letm1*-targeting shRNA lentivirus induced mitochondrial swelling and decreased mitochondrial cytochrome *b* expression in the brains of rats (Zhang et al., 2013). Furthermore, this was associated with reduced onset latency, and increased frequency and duration of pilocarpine-induced epilepsy (Zhang et al., 2013). These authors also reported lower LETM1 expression in the temporal neocortex of individuals with temporal lobe epilepsy compared with normal individuals (Zhang et al., 2013). Jiang and colleagues reported a gene-trap-based targeting of *Letm1* in mice, which resulted in early embryonic lethality for the homozygous deletion and 50% loss of the heterozygotes before E13.5 (Jiang et al., 2013). The surviving *Letm1* heterozygotes exhibited impaired brain-specific glucose metabolism and reduced ATP levels, as well as increased kainic-acid-induced seizure activity (Jiang et al., 2013).

Although the precise role of LETM1 in mitochondrial ion transport is still debated (i.e. its function as a Ca^2+^ antiporter versus K^+^/H^+^ exchange), there is growing evidence to suggest that reduced LETM1 expression can impact on different aspects of mitochondrial function (Nowikovsky et al., 2012; Nowikovsky et al., 2009). Altered mitochondrial function is strongly associated with seizure development (Folbergrová and Kunz, 2012; Kang et al., 2013). Indeed, several established mitochondrial disorders such as Kearns-Sayre syndrome and MERRF are strongly seizure-prone (Koopman et al., 2012). When considering *LETM1* haploinsufficiency, the consequent chronic mitochondrial hyperpolarisation in the context of elevated mitochondrial O_2_^−^ production and mPTP dysfunction could conceivably have catastrophic implications for normal neuronal function. Our work describes for the first time a set of mitochondrial phenotypes in WHS-patient-derived LCLs that also segregate with seizure development in the small cohort examined. This is notable because LCL C(355), which does not present with any of the aberrant mitochondrial phenotypes identified here, has a deletion that incorporates *CTBP1* and *CPLX1*, yet this individual is seizure free (Hannes et al., 2010). Furthermore, we can model some of the mitochondrial phenotypes identified in the LCLs by manipulating LETM1 expression in cell lines of neuronal (Neuro2A) and glial (T98G) origin (Fig. 6). It would be fascinating to investigate the mitochondrial phenotypes we have identified here in cells from patients presenting with seizures in the context of normal *LETM1* copy number. There is now growing evidence for a multigenic basis for seizures in individuals with deletions within 4p16.3 (Andersen et al., 2013; Misceo et al., 2012; South et al., 2007). This multigenic phenomenon is not without precedent in WHS. For example, we have recently characterised a multigenic basis for DNA replication and chromatin formation impairments in WHS (Kerzendorfer et al., 2013; Kerzendorfer et al., 2012).

The mitochondrial phenotypes we have described substantially build upon those already attributed to reduced LETM1 expression (Jiang et al., 2009; Jiang et al., 2013; Nowikovsky et al., 2012). The phenotypes described in our study are highly interdependent and likely a consequence of LETM1’s fundamental role in mitochondrial ion transport. We found elevated intracellular [Ca^2+^] and a [Ca^2+^]-insensitive mPTP in WHS LCLs with *LETM1* haploinsufficiency. Each of these could underlie or contribute to the elevated ΔΨ_mito_ and elevated mitochondrial ROS we also observed in this context (Brookes et al., 2004). Importantly, we have described these findings in the clinically relevant setting of patient-derived cells.

Our work suggests that WHS-patient LCLs could serve as a tractable model platform to investigate potential therapeutically relevant routes into mitigating against the phenotypes that we have catalogued, phenotypes that are likely relevant to seizures or additional clinical features (Liu and Schubert, 2009). For example, in some instances treatment with nigericin, an ionophore that catalyses electroneutral K^+^/H^+^ exchange in mitochondria, has been reported to reverse LETM1-dependent mitochondrial morphological changes (Dimmer et al., 2008; Nowikovsky et al., 2004; Nowikovsky et al., 2009). Nevertheless, we found that treatment of WHS LCLs with nigericin (2 μM) for up to 24 hours had no impact on mitochondrial O_2_^−^ levels (not shown).

We believe that our data provide supportive associative evidence for a contribution of reduced LETM1 expression and consequent mitochondrial dysfunction to the clinical presentation of WHS. Wider implications of LETM1-dependent mitochondrial dysfunction outside of seizures could include, for example, impacts upon optimal motor and muscle function with relevance to hypotonia in WHS. Furthermore, considering the high energy demands of the brain, LETM1-dependent mitochondrial dysfunction could impact upon other aspects of optimal neuronal function that might be relevant to intellectual disability herein. Collectively, our findings underscore the dramatic and newly identified impacts upon mitochondrial function that a modest 50% reduction in LETM1 expression has in WHS-patient-derived LCLs.

## MATERIALS AND METHODS

### Cell culture

Lymphoblastoid cell lines (LCLs) were cultured at 37°C in humidified incubators with 5% CO_2_ in RPMI 1640 supplemented with 2 mM L-glutamine, 500 U/ml penicillin, 50 μg/ml streptomycin and 15% fetal calf serum. The MERFF (myoclonus epilepsy associated with ragged-red fibres) LCL was obtained from the Coriell Cell Repository (Camden, NJ, USA). The line, GM11907, has a mutant tRNA-Lys, A>G transition at mtDNA nucleotide pair 8344/WT. Neuro-2A (N2A) cells were maintained in Dulbecco’s modified Eagle medium supplemented with 2 mM L-glutamine, 500 U/ml penicillin, 50 μg/ml streptomycin and 10% fetal bovine serum. T98G human glioblastoma cells were cultured in minimum essential medium supplemented with 10% fetal bovine serum, 1% non-essential amino acid (NEAA) and 1% sodium pyruvate, 2 mM L-glutamine, 500 U/ml penicillin and 50 μg/ml streptomycin.

### Antibodies

Anti-α-tubulin (T1568) was obtained from Sigma-Aldrich (Poole, Dorset, UK). Anti-hexokinase 1 (2024, C25C4) was obtained from Cell Signaling Technology (NEB, Hitchin, Hertfordshire, UK). Anti-β-tubulin (sc-9104), anti-MCM2 (sc-9839) and anti-LETM1 (sc-271232) were from Santa Cruz (Insight Biotec Ltd, Wembley, Middlesex, UK).

### Extract preparation

#### Urea-based whole cell extracts (WCEs)

Cell pellets were washed in PBS then lysed in 50–100 μl of urea-based lysis buffer (9 M urea, 50 mM Tris-HCl at pH 7.5 and 10 mM 2-β-mercaptoethanol), followed by a 12-second sonication at 30% amplitude. Protein concentration was determined using the Bradford Assay. Samples were then stored at −20°C or immediately boiled in 2× SDS-loading buffer (5% SDS, 10% glycerol, 10% 2-β-mercaptoethanol, 125 mM Tris-HCl, pH 6.8 and 0.2% bromophenol blue) and loaded onto SDS-PAGE gels.

### Mitochondrial isolation and extracts

Mitochondrial isolation was performed using the Miltenyi Biotec (Bisley, Surrey, UK) Mitochondrial Isolation Kit (Cat. no.: 130-094-532) according to the manufacturer’s instructions with slight modification as indicated. 10^7^ cells were lysed using the lysis buffer provided and homogenised using a glass mini-strokes homogenizer, with 10–15 strokes per sample, on ice. Lysate was incubated with anti-TOM22 magnetic microbeads for 1 hour at 4°C with gentle shaking. An LS column was placed in the magnetic field of a QuadroMACS separation unit and the lysate was then applied to the LS column. Once the lysate had run through, the column was washed 3× with the supplied separation buffer before being removed from the magnetic field and placed onto a 1 ml collection tube. The magnetically retained mitochondria were eluted in 1 ml of the supplied separation buffer and subsequently centrifuged at 13,000 ***g*** for 2 minutes to pellet mitochondria. The mitochondrial pellet was resuspended in 60 μl of urea lysis buffer and sonicated for 15 seconds at 30% amplitude. 0.1–2 μl (for LETM1) or 8 μl (for hexokinase 1) of mitochondrial extract was then immediately boiled in 2× SDS-loading buffer (5% SDS, 10% glycerol, 10% 2-β-mercaptoethanol, 125 mM Tris-HCl, pH 6.8 and 0.2% bromophenol blue) and loaded onto SDS-PAGE gels.

### Mitochondrial transition pore (mPTP) analysis

The mPTP kit (Life Technologies, Paisley, UK, Cat no.: M34153) was used according to the manufacturer’s instructions. LCLs were re-suspended in pre-warmed Hanks Balanced Salt Solution with Ca^2+^ at a final concentration of 1×10^6^ cells/ml. 3×1 ml aliquots were prepared per cell line (tube 1,2,3). 10 nM calcein AM was added to each tube, 400 μM CoCl_2_ was added to tubes 2 and 3 and 500 nM ionomycin was added to tube 3. Samples were incubated for 15 minutes at 37°C, protected from light. Cells were pelleted by centrifugation, re-suspended in 500 μl PBS and filtered into FACS Falcon tubes (Becton Dickinson, Oxford, UK) for flow cytometry analysis using a Becton Dickinson (BD) FACS Canto. Samples were analysed using 488 nm excitation and emission filters appropriate for fluorescein with BD FACS Diva software. A sample without added reagents was used for instrument set up.

### MitoTracker probes

LCLs were pelleted by centrifugation and resuspended in pre-warmed growth medium containing 250 nM MitoTracker-Red (M7512) or MitoTracker-Green (M7514) or MitoTracker-SOX (M36008), all of which were obtained from Molecular Probes, Life Technologies (Invitrogen, Paisley, UK). Cells were incubated for 15 minutes under growth conditions, protected from light. After treatment, LCLs were washed once in 1× PBS. Cells were then re-suspended in 500 μl PBS and filtered into FACS Falcon tubes for immediate flow cytometry analysis. Adherent cells (i.e. T98G and Neuro2A) were incubated with 250 nM MitoTracker probe for 15 minutes and washed once in 1× PBS. Cells were then trypsinised or detached using a cell scraper and filtered into FACS Falcon tubes for flow cytometry analysis. All data was collected using a BD FACS Canto Flow Cytometer (Oxford, UK) and analysed with BD FACS Diva software.

### Total intracellular ROS determination

Cells were treated with CellROX-Red (Life Technologies, Paisley, UK, Cat no.: C10422) at a final concentration of 5 μM and incubated for 30 minutes at 37°C. The medium was then removed and cells were washed three times with PBS. Cells were then filtered into BD FACS Falcon tubes and analysed by flow cytometry using 640/665 nm excitation/emission filters. Data was collected using a BD FACS Canto flow cytometer (Oxford, UK) and analysed with the BD FACS Diva software.

### Intracellular calcium determination

LCLs were incubated with 10 μM Calcium 1-AM probe (Life Technologies, Paisley, UK, Cat. no.: C3012) for 20 minutes under normal growth conditions. Cells were washed then re-suspended in 500 μl PBS and filtered into BD FACS Falcon tubes (Oxford, UK). Data was collected using a BD FACS Canto and subsequent analysis was performed using the BD FACS Diva software with 506/531 excitation/emission filters.

### siRNA knockdowns

#### Mouse *Letm1* (Neuro2A cell line)

ON-TARGETplus SMARTpool siRNAs against *Letm1* (L-049478-01-005, mouse) were obtained from Thermo Fisher Scientific (Loughborough, UK) and re-suspended in DPEC-treated water to a final concentration of 5 nmol. Transfections were performed using 1×5 μl siRNA against *Letm1* in the presence of 5 μl MetafectenePro (Cambio, Cambridge, UK). Cells were harvested 48 hours post-transfection. Sense target sequences are: (1) 5′-AGGUAGACAACAAGGCGAA-3′; (2) 5′-CCAACAACUUCCUGC -GUUU-3′; (3) 5′-CUAAAUAGUCGGGUGACAUA-3′; (4) 5′-CUGC -CUAAUUCAUGAGUAA-3′.

#### Human *LETM1* (T98G cell line)

Stealth siRNAs (Life Technologies, Paisley, UK) were designed against the 3′UTR region of human *LETM1* using the BLOCK-IT™ RNAi designer (Invitrogen Life Technologies, Paisley, UK). Oligos were re-suspended in 1 ml of DPEC-treated water to a final concentration of 20 nmol. Transfections were performed with 1× 5 μl siRNA against *LETM1* in the presence of 5 μl MetafectenePro (Cambio, Cambridge, UK). Cells were harvested 24 hours post-transfection. LETM1 3′UTR target sequence is: 5′-CCACAG -AAUCGUGUCUGGAUCCACA-3′.

### *LETM1* overexpression

T98G human glioblastoma cells were transfected with 2 μg plasmid encoding *LETM1* (pCMV6-XL4) purchased from Origene, in the presence of 5 μl MetafectenePro (Biontex) under normal growth conditions. Cells were harvested 24 hours post-transfection and prepared for flow cytometry or western blot analysis as described above.

### *LETM1* complementation

T98G cells were transfected with 5 μl siRNA against *LETM1* in the presence of 5 μl MetafectenePro (Biontex) + 2 μg of *LETM1-*expression vector. The vector, pCMV6-XL4 containing human *LETM1* (NM_012318), was obtained from Origene (Insight Biotec Ltd, Wembley, Middlesex, UK, Cat. no.: sc115448). At 24 hours post-transfection, cells were incubated with 250 nM MitoSOX for 15 minutes and processed as described above.

## Supplementary Material

Supplementary Material
