# Supplementary material for: LETM1 haploinsufficiency causes mitochondrial defects in cells from humans with Wolf-Hirschhorn syndrome: implications for dissecting the underlying pathomechanisms in this condition
Source: Dis Model Mech. 2014 Mar 13;7(5):535–45. doi: 10.1242/dmm.014464 (PMC4007405; doi:10.1242/dmm.014464)
Supplement: Supplementary Material [file supp_7_5_535__index.html]

LETM1 haploinsufficiency causes mitochondrial defects in cells from humans with Wolf-Hirschhorn syndrome: implications for dissecting the underlying pathomechanisms in this condition — Supplementary Material 

# *LETM1* haploinsufficiency causes mitochondrial defects in cells from humans with Wolf-Hirschhorn syndrome: implications for dissecting the underlying pathomechanisms in this condition

## DMM014464 Supplementary Material

**Files in this Data Supplement:**

- **Supplementary Material**
