# Supplementary material for: LETM1 haploinsufficiency causes mitochondrial defects in cells from humans with Wolf-Hirschhorn syndrome: implications for dissecting the underlying pathomechanisms in this condition
Source: Dis Model Mech. 2014 Mar 13;7(5):535–45. doi: 10.1242/dmm.014464 (PMC4007405; doi:10.1242/dmm.014464)
Supplement: Supplementary Material [file supp_7.5.535_DMM014464.pdf]

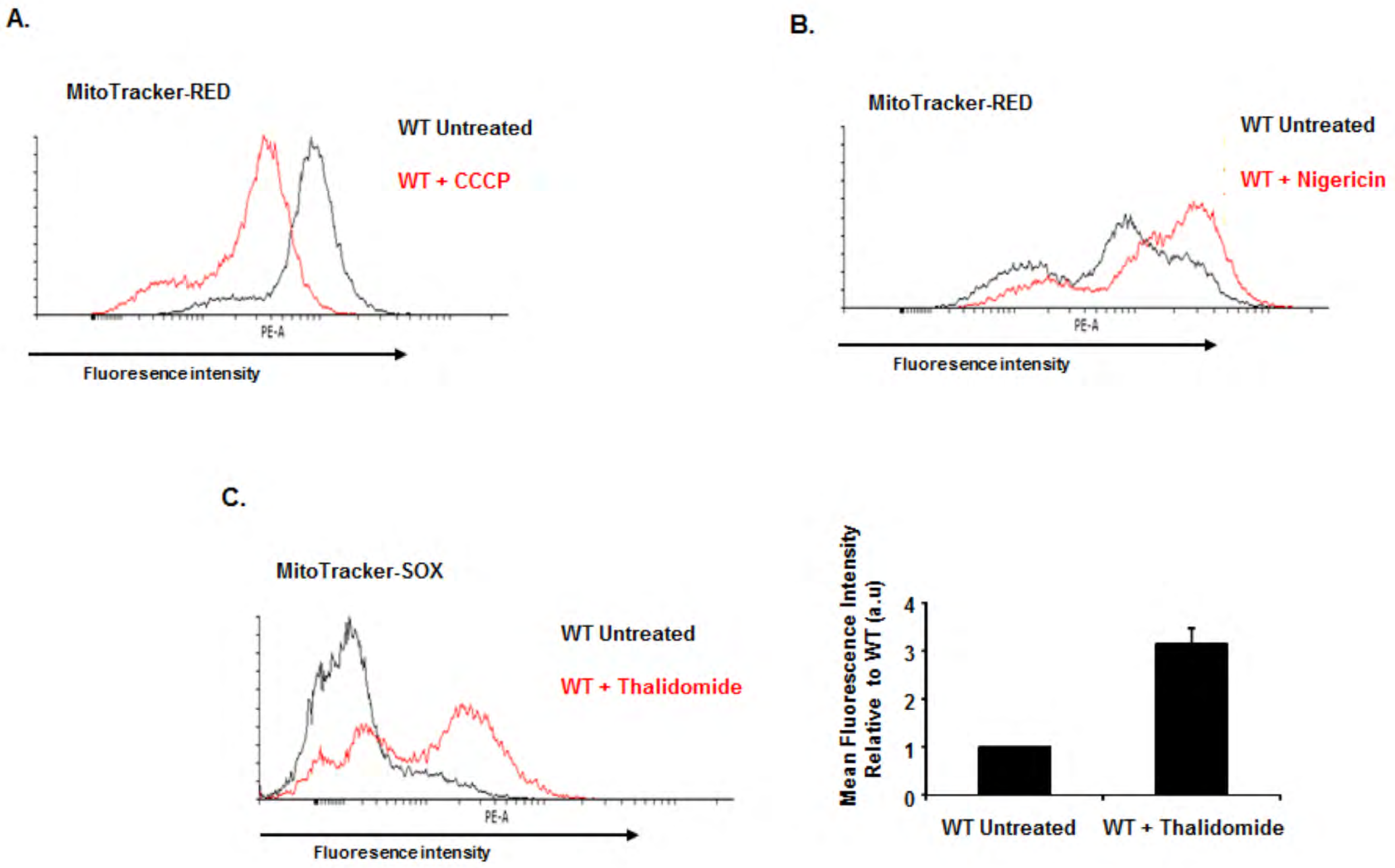

Supplementary Fig. 1. (A) MitoTracker-RED profile (PE-A mean fluorescence intensity) of wild-type (WT) LCLs either untreated or treated with Carbonyl cyanide m-chlorophenyl hydrazone (CCCP), an electron transport chain uncoupler, at 50μM for 15mins (WT + CCCP). Note the leftward shift in the profile (decreased fluorescence) indicative of depolarization following treatment with CCCP (profile shown in red). (B) MitoTracker-RED profile (PE-A mean fluorescence intensity) of wild-type (WT) LCLs either untreated or treated with Nigericin, a hyperpolarizing agent, at 2μM for 1hr (WT + Nigericin). Note the rightward shift in the profile (increased fluorescence) indicative of hyperpolarization following treatment with Nigericin (profile shown in red). (C). A contributing pathomechanism of the teratogenicity of Thalidomide is thought to be as a result of its role in generating reactive oxygen species (Parman et al., 1999; Vargesson, 2009). Treatment of wild-type (WT) LCLs with Thalidomide (500μM for 2hrs) clearly induces an approx. 3-fold increase in MitoTracker-ROS fluorescence (profile shown in red), compared to untreated WT LCLs. The graph on the right shows the relative fold increase (mean fluorescence intensity) following Thalidomide treatment, compared to untreated. Data represents the mean ± s.d. of three independent experiments (PE-A: area under the curve).
